# Real-Time Detection of LAMP Products of African Swine Fever Virus Using Fluorescence and Surface Plasmon Resonance Method

**DOI:** 10.3390/bios12040213

**Published:** 2022-04-03

**Authors:** Hao Zhang, Yuan Yao, Zhi Chen, Wenbo Sun, Xiang Liu, Lei Chen, Jianhai Sun, Xianbo Qiu, Duli Yu, Lulu Zhang

**Affiliations:** 1 College of Information Science and Technology, Beijing University of Chemical Technology, Beijing 100029, China; zh1632414919@163.com (H.Z.); yy_xyyyyy@163.com (Y.Y.); xbqiu@mail.buct.edu.cn (X.Q.); dyu@mail.buct.edu.cn (D.Y.); 2 Shandong Key Laboratory of Animal Disease Control and Breeding, Institute of Animal Science and Veterinary Medicine, Shandong Academy of Agricultural Sciences, Jinan 250100, China; charleschenzhi@163.com; 3 Aerospace Information Research Institute, Chinese Academy of Sciences, Beijing 100094, China; sunjh@aircas.ac.cn; 4 Shandong Provincial Key Laboratory of Animal Resistance Biology, College of Life Sciences, Shandong Normal University, Jinan 250014, China; chenlei@sdnu.edu.cn

**Keywords:** African swine fever virus (ASFV), loop-mediated isothermal amplification (LAMP), surface plasmon resonance (SPR), fluorescence detection

## Abstract

African swine fever (ASF) is a swine disease with a very high fatality rate caused by a complex double-stranded DNA virus. The fluorescence PCR detection method is widely used for virus nucleic acid detection. Surface plasmon resonance (SPR) is a label-free and real-time detection method, unlike the fluorescence PCR detection method. In this research, we detected the loop-mediated isothermal amplification (LAMP) products of the African swine fever virus by using the SPR and fluorescence methods separately and simultaneously. By comparing the positive and negative control results, we found that the SPR response unit is completely different before and after the LAMP process. In addition, the fluorescence results on a chip showed that with an increase in the concentration of the sample, the cycle threshold (CT) value decreased, which is consistent with commercial instruments. Both the decline rate of the SPR response unit and the CT value of the fluorescence realized were used to distinguish the positive control from the negative control and water, which indicates that the SPR method can be combined with fluorescence to detect LAMP products. This research provides a label-free and simple method for detecting LAMP products.

## 1. Introduction

African swine fever (ASF) has the features of acute and high contagiousness and a fatality rate of 100%. The subclinical infection of African wild boars, such as bush pigs and warthogs, can last for months or even years. In the last decade, the pig industry in about 50 countries around the world has been affected by this virus, making this industry precarious [1,2]. The African swine fever virus is a kind of complex double-stranded DNA virus with a size of 170–190 kbp [3]. Therefore, regular virus detection in pigs is a reasonable way to avoid risks.

The hemadsorption test, virus isolation, and real-time polymerase chain reaction (real-time PCR) are widely used methods for ASFV diagnosis [4]. Hemadsorption tests and virus isolation are reliable methods for virus detection, but their operations are complex and time consuming, which makes them unsuitable for rapid detection. Real-time PCR is recognized as the most sensitive and reliable method. However, it contains complex temperature-changing devices, which is not conducive to field rapid detection application.

Loop-mediated isothermal amplification (LAMP) is a technique for isothermal amplification at 60 °C–65 °C. Its strong points are its convenient operation, high efficiency, high specificity, and short reaction time [5]. It is suitable for detection in complex environments and can be well applied in the detection of African swine fever. At present, the real-time fluorescence detection method is widely used in LAMP. The following are some application examples: Jiang [6] used LAMP techniques to establish a rapid method for the detection of African swine fever. Xing [7] completed the LAMP and fluorescence detection of Zika viruses on a small microfluidic chip. A capillary-array microsystem with integrated DNA extraction, loop-mediated isothermal amplification, and fluorescence detection was used and, based on this system, successfully achieved the detection of Mycobacterium tuberculosis [8]. However, the fluorescence detection method needs to label the sample, and fluorescence bleaching and quenching especially lead to inaccurate detection results.

The use of surface plasmon resonance (SPR) technology is a label-free method used to measure refractive index changes on the surface of a sensor chip due to biological interactions caused by optical principles. SPR technology has the advantages of being simple to operate and label free as well as allowing real-time detection [9,10,11,12]. Bai [13] used a portable SPR biosensor to detect the H5N1 virus, but the detection concentration range has certain limitations. Wang [14] reported a label-free detection of single virus particles in solution after using surface plasmon resonance. Nguyen [15] reported a sandwich SPR biosensor detection method that can quickly and accurately detect the whole H5N1 avian influenza virus. Wang [16] developed an SPR biosensor platform based on fast and sensitive intensity modulation. It was used to detect H7N9, and its detection limit was about 20 times that of ELISA. Yoo [17] developed a reusable magnetic SPR sensor chip that can be used to repeatedly detect the H1N1 virus. However, the above SPR detection methods are based on the principle of immune interactions between biomolecules and are not combined with nucleic acid amplification and fluorescence detection.

The use of multiple testing methods together often leads to better results [18]. The combination of these technologies should improve detection sensitivity and specificity. We have realized the synchronous detection of microspheres and A549 cells by using the SPR and fluorescence methods with a homemade microscopic imaging system [19]. This system is complex in size and cannot realize the rapid amplification and detection of the virus on site. For this paper, a portable SPR imaging biosensor of the classic Kretschmann prism structure was designed to detect LAMP products [20]. By detecting the SPR response unit before and after LAMP, it was found that the decline rate of the SPR response unit for the positive sample can be used as an indication of the LAMP result. The independent fluorescence detection system was integrated with the heating module, and a microfluidic chip was used to complete the LAMP and fluorescence detection on the chip. The higher the concentration of the positive control was, the lower the CT response unit of fluorescence detection became. Finally, these two systems were combined to complete the LAMP on the chip, and the SPR and fluorescence detection methods were used to simultaneously detect the LAMP. This paper established a faster, more accurate, label-free, and highly sensitive detection method.

## 2. Materials and Methods

### 2.1. Materials and Reagent

The ASFV LAMP kit (HaiGene) and the ASFV LAMP kit (Quicking Biotech, Shanghai, China) were used in this paper. According to the requirements of the kits, 2 μL of different concentrations of positive control or negative control were added to 20 μL of reaction solution and enzyme mixture to configure different reaction mixtures. This took one minute per amplification cycle. The other reagents used in the experiments were purchased from HyperCyte Biomedical Co., Ltd., Beijing, China.

### 2.2. Systems of Detection

#### 2.2.1. SPR Detection System

**Figure 1:**
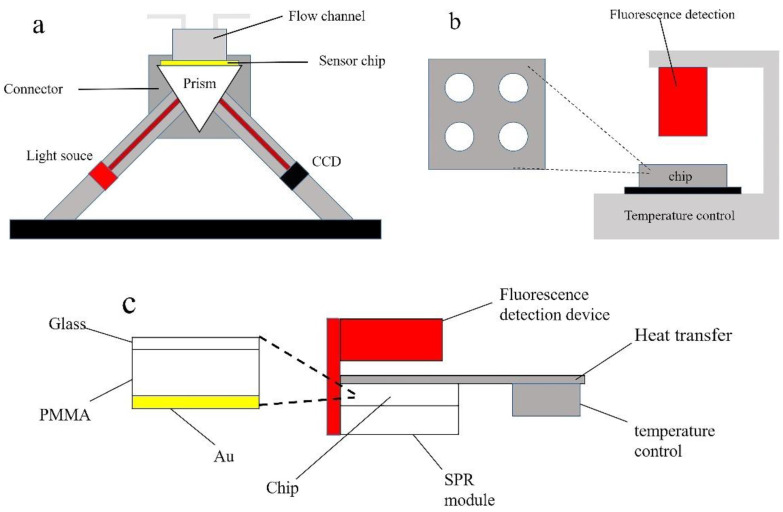
(**a**) Schematic of independent SPR system composed of light source, CCD camera, flow cell, prism and gold layer. (**b**) Schematic of the independent fluorescence detection system consisting of heating module and microfluidic chip. (**c**) Schematic of the integrated device of fluorescence detection, SPR module, temperature, and chip.

The schematic diagram of surface plasmon resonance (SPR) biosensors is shown in Figure 1a, which was designed by us [21]. A semiconductor red laser with a wavelength of 633 nm was used as a light source, and a CCD camera was used as a light-receiving device. The light source and CCD camera were symmetrical around the prism, and we could change the angle of the light source and prism by motor movement. The angle scan was used to find the SPR resonance angle at which the device could then be fixed for the experiment, and the CCD camera was used for the detection of the real-time SPR response unit. The chip and flow cell were located above the prism, and the liquid passed through the flow cell onto the surface of the chip.

#### 2.2.2. Fluorescence Detection System

The independent fluorescence detection system integrated with the heating module and microfluidic chip (abbreviated as the independent fluorescence system) was used for LAMP and fluorescence detection on the chip. The temperature control module was located at the bottom of the whole integrated device and was mainly used to provide the temperature required by the LAMP. The chip was fixed above the temperature control device by a metal fixing device so that the heat could be efficiently transferred to the chip. There were 4 reaction chambers on the chip designed for this experiment, and 4 groups of control experiments could be performed simultaneously. Before the experiment, the prepared reagents were added to different chambers on the chip, and then the chip was sealed with a transparent glass sheet. The fluorescence detection module was located directly above the chip and could collect the fluorescence response unit of the solution in the chip in real time.

### 2.3. Integrated Device of SPR Sensor Chip and Heating Module

To realize the combination of LAMP, SPR detection, and fluorescence detection, we designed an integrated device as shown in Figure 1c. The LAMP chip was fixed on the prism instead of the flow cell (in Figure 1a), and the aluminum block (which does not hinder fluorescence detection) was connected to the temperature control device and the chip, acting as a heat transfer device so that the chip could reach the temperature (64 °C) required by the LAMP. The independent fluorescence detection (in Figure 1b) was located directly above the chip and fixed with the SPR detection device (in Figure 1a). It could be controlled by software for temperature adjustment, SPR detection, and fluorescence detection. The LAMP-SPR chip was mainly composed of three layers, and the adjacent two layers were bonded by double-sided tape (purchased from 3M) to avoid liquid leakage. The bottom layer was glass sputtered with Au for SPR detection. The middle layer was the subject part made of PMMA material, which was used to hold liquid and carry out LAMP. On the top was a very thin, transparent layer of glass (0.13–0.17 mm) that could cover the entire chip without affecting the fluorescence collection.

### 2.4. Experiment Procedure

#### 2.4.1. SPR Detection Procedure

To verify whether SPR can be used to detect LAMP products, the following experiments were carried out: According to the requirements of the ASFV LAMP kit (Quicking Biotech, Shanghai, China), reagents were configured. A PCR instrument was used to amplify the positive control sample for 15 cycles and 35 cycles, and the negative control sample for 35 cycles (abbreviated as pos-15, pos-35, neg-35). Biacore T100 (GE) was used to carry out the same SPR experiment. We also detected the LAMP products of different concentrations of positive control by using the SPR method. Positive nucleic acids of 0.5, 1, and 2 μL were prepared with an ASFV LAMP kit (Quicking Biotech, Shanghai, China), in which an appropriate amount of deionized water was added to form an equal volume of positive control for 2 μL. The SPR biosensor judges whether amplification occurs by detecting the change in the refractive index of the sample, so the mixed amplified sample was injected into the SPR biosensors before and after amplification to test the SPR response unit, and the difference in the response unit was calculated.

#### 2.4.2. Fluorescence Detection Procedure

To demonstrate that LAMP on a chip gives similar results to LAMP in a PCR instrument, the following experiments were performed. According to the method in Section 2.4.1, the positive control solution with volumes of 0.2, 0.5, 1, and 2 μL and the negative control with a volume of 2 μL were prepared. The PCR instrument (Bio-rad) and independent fluorescence detection system (in Figure 1b) with a microfluidic chip were used to compare the fluorescence signals of LAMP. To compare the differences between different reagents, the ASFV LAMP kit (HaiGene, Haerbin, China) was used to configure the same concentration of reagents (in Figure 1b) and complete the LAMP and fluorescence detection with the independent fluorescence detection system.

#### 2.4.3. SPR and Fluorescence Simultaneous Detection Procedure

The following experiments were used to prove the feasibility and stability of the integrated device for LAMP, SPR detection, and fluorescence detection: The mixture of the 2 μL positive control and 20 μL reaction solution and enzyme were prepared using the ASFV LAMP kit (Quicking Biotech, Shanghai, China), and then it was added to the chip and sealed. At the beginning of the experiment, we let the chip stand for 5 min to obtain the initial fluorescence and SPR response unit, and the temperature control device was used to heat the chip to 64 °C so that it started LAMP (this process took 10 min). The heating device was turned off after 50 min, and data were recorded until the device dropped to room temperature. This experiment was repeated three times.

## 3. Results and Discussion

### 3.1. SPR Detects LAMP Products

The LAMP process reduces the refractive index of the reaction solution [22]. The SPR method can theoretically be used to detect the LAMP products and judge whether LAMP has occurred by comparing the decline rate of the SPR response unit. In this paper, the response unit detected by the SPR method before amplification is defined as α, the response unit after amplification is defined as β, and the decline rate of the SPR response unit is defined as *R*.

*R* = (α − β)/α × 100% (1)

**Figure 2:**
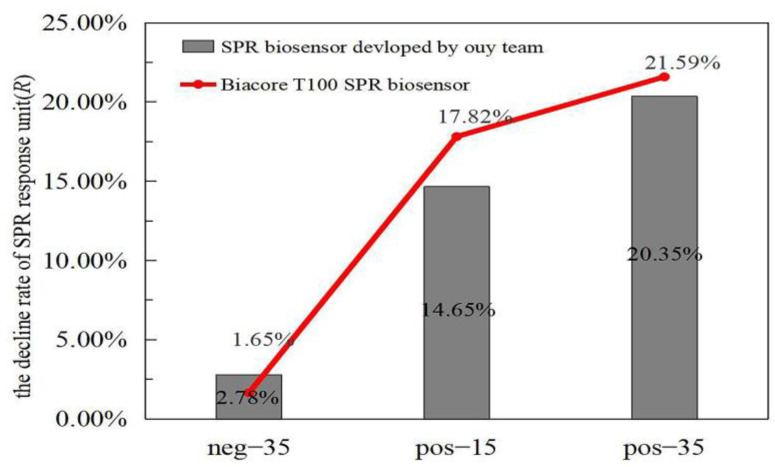
To compare the decline rates of SPR response units of the two instruments, the line graph represents the test results of Biacore T100 SPR biosensor, and the histogram represents the test results of the self-made SPR biosensor. This figure mainly compares the decline rate of SPR response unit (*R*) of positive control amplified for 15 cycles (pos−15), positive control amplified for 35 cycles (pos−35), and negative control amplified for 35 cycles (neg−35) of two instruments. The volume of the positive and negative controls in this experiment was 2 μL.

**Figure 3:**
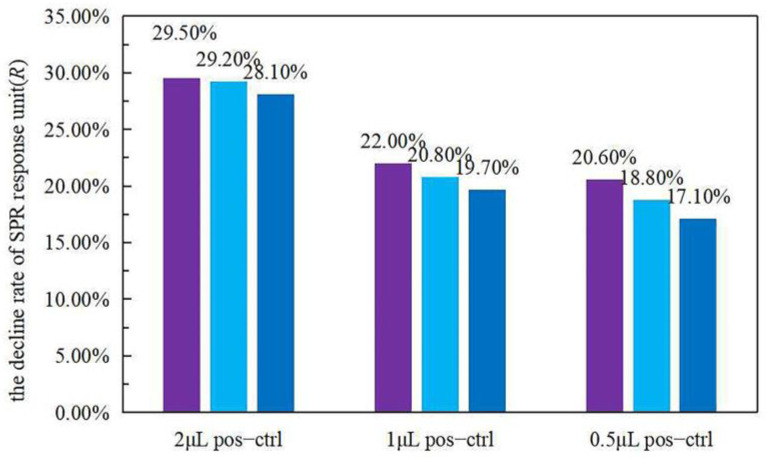
Tests were repeated three times for the decline rate of positive control (pos−ctrl) at different concentrations. It is clear that solutions with low concentrations of positive control have a small decline rate.

To prove that SPR can detect LAMP products, the SPR response units before and after amplification were detected for comparison. As shown in Figure 2, the results of both of the instruments tested show a higher rate of decline in reaction units for pos-35 than pos-15, and both were much higher than neg-35. The volume of the positive control in this experiment was 2 μL. The detection results for the different concentrations of the positive control are shown in Figure 3, from which it can be seen that lower concentrations of positive control show a lower rate of decline. These results prove that the LAMP process reduces the refractive index of the reaction solution, and the SPR method can be used to judge whether LAMP has occurred.

### 3.2. Fluorescence Detection of LAMP on a Microfluidic Chip

**Figure 4:**
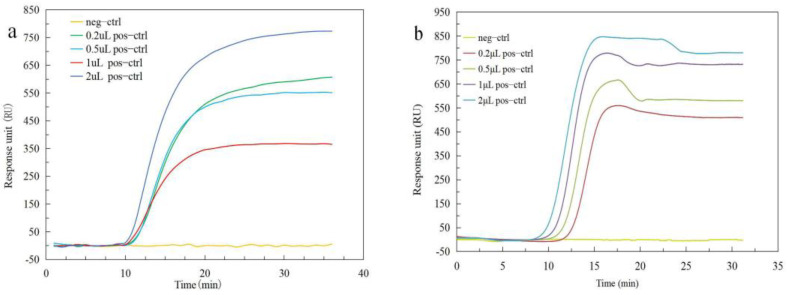

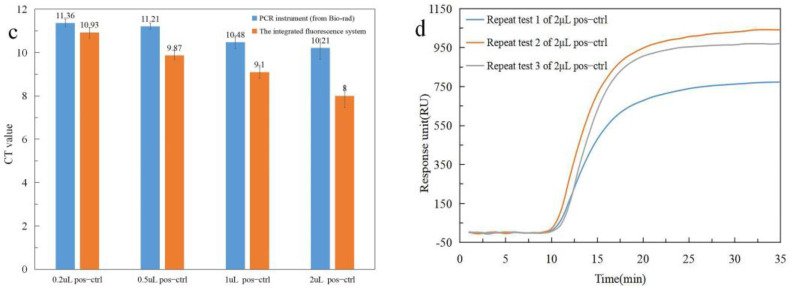
Comparing the LAMP and fluorescence detection results of the independent fluorescence system and the PCR instrument. (**a**) The fluorescence detection process of different concentrations of positive control (pos−ctrl) with PCR instrument (Bio−rad). (**b**) The independent fluorescence detection system on a chip for the same experiments as in Figure 4a. (**c**) CT values of different pos−ctrl with two instruments. (**d**) Comparison of the results of three fluorescence detections using the same concentration of reagent (positive control at a volume of 2 μL). The CT values were similar, but the final fluorescence responses were very different.

The independent fluorescence system (in Figure 1b) was used to perform LAMP on the chip, which is similar to LAMP on the PCR instrument (Bio-rad). The reaction solution of the negative control and the different concentrations of the positive control were tested in the two instruments. Figure 4a,b show the detection process and the results of the PCR instrument and our home-made fluorescence detection system, respectively. The fluorescence detection CT values of PCR and the independent fluorescence system are shown in Figure 4c. It can be seen that the amplification response unit, after 20 min in the PCR machine, did not have a linear relationship with the concentration. Therefore, the CT value was used as the basis for judging the nucleic acid concentration. The CT value changes according to the law, that is, the higher the concentration, the lower the CT value. Although their error bars will intersect slightly, this does not affect the overall trend. The result of the independent fluorescence system on-chip is the same as the trend of the PCR instrument, which also illustrates the success of the LAMP on-chip. We performed three experiments with the same reagents (positive control at a volume of 2 μL) in the standard PCR instrument, and the results are shown in Figure 4d. It can be seen that the CT values of the three experiments are similar, but the responses after 20 min are very different, which proves that the final fluorescence response cannot determine the results of LAMP. The CT values of the two kits are similar, indicating that the two kits have little effect on the experiment.

### 3.3. Simultaneous Detection of LAMP Products by SPR and Fluorescence on a Microfluidic Chip

**Figure 5:**
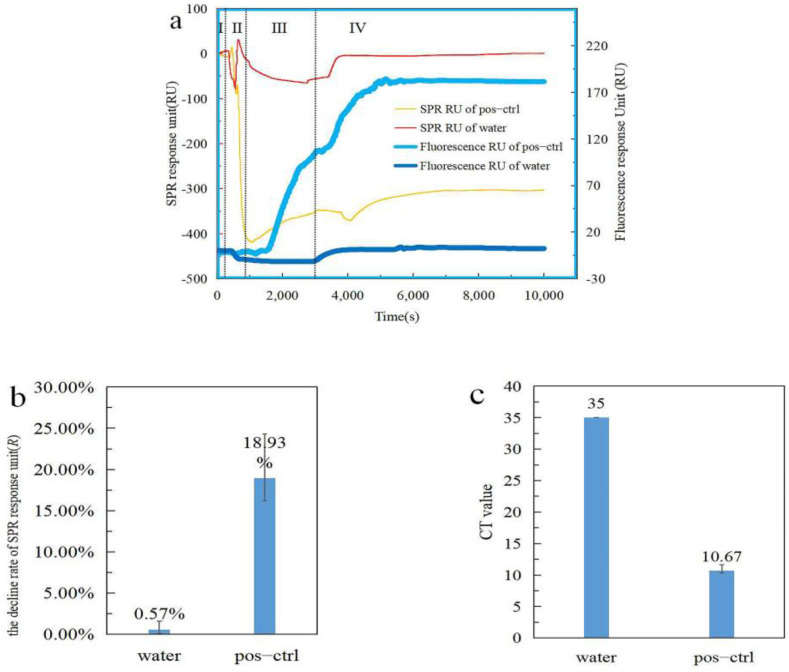
The results of simultaneous fluorescence and SPR detection in the integrated device are shown in this figure, (**b**,**c**) show the results of three replicates. The volume of positive control in this experiment was 2 μL. (**a**) The results of SPR and fluorescence response unit of water and positive control during the LAMP process; the left ordinate in this figure is the SPR response unit, and the right is the fluorescence response unit. Region I of the figure shows the process of detecting the baseline, region II is the pre−warming part, region III is the amplification part, and region IV is the process of cooling to room temperature after the amplification is completed. (**b**) The decline rate of SPR response unit of water and positive control. (**c**) CT values of fluorescence detection (because water has no fluorescence response unit, it is assumed that its CT value is 35).

LAMP reagent (Quicking Biotech, Shanghai, China) was added to the integrated device to verify whether it could be detected by SPR and fluorescence at the same time. The results are shown in Figure 5a. The volume of the positive control in this experiment was 2 μL. It can be seen that the fluorescence response unit of water did not change much. However, the fluorescence response unit of the positive control is similar to the previous experiment (data in Section 3.2). This illustrates the success of LAMP on the chip in the integrated device. Observing the SPR response unit, we found that when the temperature changes (region II in Figure 5a), both the water and the positive reference SPR response unit change sharply. After this (region III in Figure 5a), the water response unit gradually stabilizes while the positive control response unit slowly rises. This proves that the amplification reaction is taking place. After the amplification (region IV in Figure 5a), the SPR response unit begins to change sharply due to temperature changes and finally stabilizes. After stabilization, the SPR response unit of water almost returns to the initial value (region I in Figure 5a), but the value of the positive control drops greatly. When the temperature changes, the SPR response unit is very sensitive, resulting in a slight deviation in the detection of the LAMP process by the SPR. It is better to judge this using the decline rate of the SPR response unit. The decline rate of the SPR response unit and the CT values of fluorescence for the positive control and water samples are shown in Figure 5b,c. By comparing the SPR response units before and after amplification, it can be seen that the rate of response unit decline of the positive control is 18.93%, which is similar to the previous results (data in Section 3.1), whereas the response unit of water does not change much. The results of the SPR response unit also proved that the amplification was successful and that the amplification products were simultaneously detected by SPR and fluorescence. The same experiment was performed three times, which shows that the experiment is repeatable and also proves the feasibility of detecting LAMP products by SPR and fluorescence.

### 3.4. Discussion

Traditional loop-mediated isothermal amplification methods for detecting fluorescent signals in nucleic acids require fluorescent labeling, and fluorescent molecules have problems such as easy quenching and photobleaching, which affect the accuracy of the detection. In this paper, we demonstrate through the experiments of SPR signal detection before and after LAMP that the SPR method can detect the refractive index change of amplification products and that the SPR signal can be detected in real time during the amplification process. In addition, we demonstrate that the difference in the SPR refractive index signal can be measured at different amplification cycles and different concentrations of the positive control to be tested and that the amplification reagents do not need to be labeled, which can avoid the problem of fluorescence quenching that occurs with fluorescence detection techniques. However, the SPR detection method is very sensitive to temperature, and fluctuations in temperature can lead to inaccurate signals, so an accurate temperature control system is required, and this is where further development and improvement are needed. SPR and fluorescence signals are currently combined to analyze the reaction process of nucleic acid amplification, both of which can be monitored in real time, but because the fluorescence method takes a signal every minute to avoid the attenuation of fluorescence intensity, whereas the SPR signal can be monitored every second in real time (or even at a higher frequency), the combination of the two methods ensures that the amplification process is efficient and allows for a more detailed and precise analysis of the kinetic change process in amplification. In particular, the fluorescence signal rises exponentially during the stage at which the signal starts to rise, and the precision sampling of the SPR is a more accurate response to the rapid change in the amplification peak. Thus, the work in this paper on the simultaneous detection of LAMP products by two methods provides a platform and means for multidimensional information analysis of nucleic acid detection, which can be extended to the nucleic acid detection of other infectious diseases in addition to its application in the detection of African swine fever, such as detecting Corona Virus Disease 2019.

## 4. Conclusions

In this paper, LAMP products were measured separately and simultaneously by two methods. The results show that the higher the level of positive control, the lower the CT value. SPR reaction units before and after the amplification of the LAMP product can be used to determine whether LAMP has occurred. The feasibility and stability of an integrated instrument for LAMP, SPR, and fluorescence detection are demonstrated. This instrument allows for a rapid, label-free, real-time multi-detection method that can be used in complex environments. Although some issues require improvement, they provide a reference for future research.

## Data Availability

Not applicable.
